# The prevalence of Group B Streptococus recto-vaginal colonization and antimicrobial susceptibility pattern in pregnant mothers at two hospitals of Addis Ababa, Ethiopia

**DOI:** 10.1186/1742-4755-11-80

**Published:** 2014-12-05

**Authors:** Zufan Lakew Woldu, Tatek Gebreegziabher Teklehaimanot, Sisay Teklu Waji, Mahlet Yigeremu Gebremariam

**Affiliations:** Department of Gynecology and Obstetrics, Addis Ababa University, School of Medicine, P.O. BOX: 27954/1000, AA, Addis Ababa, Ethiopia; Medical Laboratory Technology, Addis Ababa University, School of Medicine Tikur Anbessa Hospital, Addis Ababa, Ethiopia

**Keywords:** Group B streptococcus (GBS), Recto-vaginal colonization, Prevalence

## Abstract

**Background:**

Group B streptococcus (GBS) has been implicated in adverse pregnancy outcomes. GBS recto-vaginal colonization rates significantly vary among different communities and geographic locations. Limited data is available on the prevalence and effects of GBS recto-vaginal colonization among pregnant mothers in developing countries like Ethiopia.

**Objective:**

To assess the prevalence of GBS recto-vaginal colonization among near term pregnant mothers and the antimicrobial susceptibility pattern of the isolates.

**Methods:**

A cross sectional descriptive study was conducted on pregnant mothers at gestational age of 35–37 weeks attending Ante Natal Clinics at Ghandi Memorial (GMH) and Tikur Anbessa Specialized Hospital (TASH) in Addis Ababa. Samples from lower genital tract and rectum were collected and cultured for GBS on CHROM agar Strep B.

**Results:**

Twenty two of the 300 pregnant mothers (7.2%) studied were found to have positive GBS recto-vaginal culture. Twelve isolates (55%) were sensitive to penicillin while 20 (91%) were sensitive to ampicilline. All isolates except one were sensitive to Erythromycin.

**Conclusion:**

The study showed recto-vaginal GBS colonization among near term pregnant mothers is reasonably high in our community calling for the need to screen mothers near term and provide appropriate antimicrobial prophylaxis to prevent potential adverse maternal and neonatal outcome.

## Introduction

Pregnant women colonized by Group B Streptococcus (GBS) are known to be at an increased risk of adverse obstetric outcomes. GBS is also known to infect the newborn and hence increase the neonatal morbidity and mortality[1–3]. Epidemiological studies have revealed that pregnant women colonized with GBS are 25 times more likely to deliver infants with early onset GBS disease than women with negative prenatal cultures[4, 5].

Chemoprophylaxis remains the most effective means to prevent maternal and neonatal infections. Penicillin, ampicillin, erythromycin and clindamycin are drugs of choice for antibiotic prophylaxis against GBS. However reports of emerging resistance to theses drugs have made susceptibility study an important component of GBS control strategy. Rate for neonatal invasive disease both early and late onset is between 1.7 and 3.3 per 1000 live births in the USA and 0.2-0.6 per 1000 live births in Europe, Canada and Israel[2, 3]. Studies in USA showed case fatality rates for the early onset GBS disease to be 5 to 20%[6]. Infants born to heavily recto-vaginal GBS colonized women were also at an increased risk of neonatal sepsis[6]. Although excellent data are available from the developed world, there is little data on GBS recto-vaginal colonization and associated maternal and neonatal complications in developing countries.

One study in Ethiopia, Gondar College of Medical Sciences in 1987, involving 200 postpartum women, and 80 newborn infants reported GBS recto-vaginal colonization rate of 9% in postpartum women and GBS oro-pharyngeal colonization rate of 5% in the newborn infants[7].

In a study conducted in Peru, Lima, the overall prevalence of recto-vaginal colonization of GBS was reported to be 8%. GBS was isolated more frequently from the vaginal than the rectum[8].

Rates of GBS colonization vary widely throughout the globe. Culture methods, including the number and type of sites cultured and type of medium used, have accounted for some of this variations[8]. Despite the differences in technique, real regional variation exists. High prevalence rates have been reported from the United States (15-25%), Jordan (30%) and Gambia (22%)[2, 9, 10]. One study in Mexico City showed prevalence rate of 4%[11]. Low prevalence rates have also been reported in Italy (6.6%) and Turkey (8.7%)[12, 13]. A report from Iran revealed 9.1% GBS colonization rate among pregnant mothers. In addition to regional differences, some investigators have also reported racial differences in GBS colonization rates[14].

Similar studies done on pregnant women in Tanzania and Malawi revealed a higher GBS colonization rate of 23% and 16.5% respectively[15, 16].

The magnitude of the problem and its public health importance was appreciated by academic institutions and organizations and strategies to prevent related morbidities and mortalities was launched. After reviewing scientific data and collecting expert opinions, American College of Obstetricians and Gynecologists (ACOG) and Centers for Disease Control (CDC) in 1996 developed guideline on prevention of perinatal GBS disease and that was revised again in 2002[17–19].

This study was designed to evaluate the prevalence of recto-vaginal colonization of GBS in pregnant mothers near term attending Ante Natal Clinics (ANC) in two teaching Hospital in Addis Ababa, identify associated socio demographic and obstetric risk factors and assess the antimicrobial sensitivity pattern of the isolates.

## Materials and methods

A cross sectional descriptive study was conducted on pregnant mothers at gestational age of 35–37 weeks, who were not taking any antibiotics, attending ANC at Gandhi Memorial Hospital and Tikur Anbassa Specialized Hospital for a period of three months in 2010. These hospitals are referral hospitals providing comprehensive ante-partum, intra-partum and post-partum care. Nearly 8000 mothers deliver in these hospitals annually.

A single proportion formula with a standard deviation, Z of 1.96, a degree of precision f 0.05, was used to calculate sample size. Sample size n = 300 was calculated using P = 9% from study done in Gondar, Ethiopia[7]. After the hospitals ethics board permission was obtained, mothers who fulfilled the inclusion criteria and who were willing to participate in the study were selected by simple quota sampling technique until a total of 300 mothers were recruited. Verbal consent was secured after reading a written consent (because of low literacy rate) that was approved by the department of obstetrics and gynecology research and publication committee and after explaining the objective of the study and its potential benefits to the mothers and their neonates. IRB: Addis Ababa University, college of health sciences Institutional Review Board.

Data was collected through interview and review of the ANC charts. Analysis was done using the SPSS stat software. Samples from the upper vagina and rectum were collected for each mother using sterile applicator and were transported at room temperature using Amies transport media. It was then inoculated on to CHROM agar sterp B plate (made by Chroagar, Paris) within 1–6 hours of collection. The plates were incubated at 37 degree Celsius for 24 hrs. After 24 hrs, only positive cultures were analyzed and culture medias with no growth were discarded. Isolates were identified based on colonial morphology, catalase reaction, CAMP test and strept B latex test (made by Remel, Canada). Antimicrobial sensitivity to commonly used antibiotics like penicillin (R < 11 mm, S > 22 mm), ampicillin (R < 20 mm, S > 29 mm), erythromycin (R < 13 mm, S > 18 mm) and clindamycin (R < 15 mm, S > 19 mm) was done by blood agar, using agar diffusion test technique. Results of those mothers with positive GBS culture were communicated to the treating physician for prophylactic measures, as there is no GBS prophylaxis protocol in place in Ethiopia.

## Results

Twenty two (7.3%) among the 300 pregnant women screened were colonized by S. aglactae. As shown on Table 1, twenty mothers with GBS colonization positive cultures are in the moderate income group while two were in the lower income group. Eleven (50%) mothers were primigravida while 8 (36.5%) had one and 3 (13.5%) had two or more prior pregnancies. Culture for GBS was positive in 10 mothers in the age range of 20 to 25 years, in seven mothers 26 to 30 years, and three mothers 31 to 35 years while one was below 20 years and one above 36 years. In vitro antimicrobial susceptibility pattern of GBS isolates is shown in Table 2.

**Table 1:** **Prevalence of recto vaginal GBS colonization and socio-demographic characteristics, Addis Ababa, Ethiopia 2010**

Recto vaginal GBS	Culture positive	Culture negative
	N (%)	N (%)
	22 (7.3%)	278 (92.7%)
Age (Years)		
< 20	1	8
20-35	20	252
>35	1	20
Total	22	278
Family income (monthly, USD)		
<10	2	29
10-50	11	122
51-100	9	119
>100	0	8
Total	22	278
Parity		
Nulli para	11	91
One	8	122
Two or more	3	65
Total	22	278

**Table 2:** **GBS in vitro antimicrobial sensitivity pattern, Addis Ababa, Ethiopia, 2010**

Antimicrobial	Sensitive	Intermediate	Resistant	Total
	N (%)	N (%)	N (%)	
Clindamycin	19	1	2	22
Erythromycin	20	-	2	22
Ampicillin	20	1	1	22
Penicillin	12	2	8	22

## Discussion

GBS colonization rate of 7.3% in term pregnancies seen in this study is higher than 6.6% reported from Italy but lower than many reports in the literature[2, 9, 12, 13, 15, 16]. The reason for the varying results may be attributed to the fact that GBS maternal colonization varies from place to place. Other factors that may have contributed to this variation include socio-economic factors, variation in clinical practices, method of sample collection and the techniques. Besides, ethnic and genetic factors might play a role in variation of the rates of infection with GBS[6]. In this study maternal GBS colonization rate was seen more among the primigravidae and second gravidas. This was consistent with the findings in other studies[5]. It is also suggested that this greater GBS colonization often observed among the primigravidae and the second gravida has epidemiological implications in terms of maternal complications and neonatal infections and policy issues for introducing preventive interventions[5, 18].

However, the reason for this parity–specific group B streptococcal susceptibility is not clearly understood. Overall, 86.5% of the women with GBS are primigravida or nulliparas. This finding is consistent with findings from another study that showed 88.9% of women with positive GBS cultures to have three or fewer pregnancies[7].

GBS colonization has significant correlation with the risk of perinatal infection[8]. CDC in 1996 also found that mothers who are carriers of GBS have 50% chance of infecting their babies before or during birth[18]. The finding of more positive isolates in lower parity in this study indicate that infants born to the primigravida and second gravida mothers, who are carrying GBS, may be at risk of developing GBS neonatal disease. Of the GBS positive cases studied, 77.3% were women of 20 to 30 years of age. This is expected as this is the age of increased sexual activity and GBS organisms are known to be sexually transmitted[4].

The GBS organisms isolated in this study showed higher susceptibility to ampicillin and erythromycin while significant number showed resistance to penicillin. This finding differs from the study in Calabar, which showed 100% sensitivity to penicillin[7]. This may be due to the wide and non prescription use of penicillin in our community because of weak drug control mechanism. However the finding has significant clinical implication regarding the use of penicillin in the treatment of GBS related infections both in mothers and their neonates. Ampicillin has been preferred by numerous investigators as a drug of choice because of its safety during pregnancy and its broader spectrum compared to penicillin.

Though this study has limitations and weaknesses in terms of small sample size, non probability quota sampling method and neonates are not included in the study to assess the neonatal infection rate compared to other studies, the result of this study will help clinicians and policy makers to understand the magnitude of the problem and plan a uniform protocol based on antibiotic sensitivity pattern. The findings in this study indicate the need for future large scale, preferably cohort, study in Ethiopia.

## Conclusion and recommendation

The prevalence of GBS colonization seen among mothers attending ANC at two referral hospitals in Addis Ababa, Ethiopia, though is lower compared to other studies in similar settings in Africa, indicates the need to screen mothers near term and plan appropriate intervention. Further large scale study to ascertain the prevalence of GBS colonization among pregnant mothers at different gestational age and the effects on the outcome of pregnancy, both maternal and neonatal should be conducted to introduce national guideline.

## Authors’ information

ZW is an associate professor at Addis Ababa University, School of Medicine, Department of Obstetrics and Gynecology. She is involved in teaching and service de4livery at the Hospital. She was a Department head for three years and also served as Dean of the School of Medicine for another three years.

TT is an active member of the medical laboratory technology at Addis Ababa University, School of Medicine. He is involved in teaching and also give service in the Hospital Laboratory.

SW is an assistant professor of Ob-Gy at Addis Ababa University, School of Medicine. He is currently involved in teaching under graduates and post graduates in the department. Was involved in several Obstetric and gynecology research activities.

MG is an assistant professor of Ob-Gy at Addis Ababa University, School of Medicine. She is currently involved in teaching under graduates and post graduates in the department.
